# 2-oxoglutarate-dependent dioxygenases: A renaissance in attention for ascorbic acid in plants

**DOI:** 10.1371/journal.pone.0242833

**Published:** 2020-12-08

**Authors:** Asaad M. Mahmood, Jim M. Dunwell

**Affiliations:** 1 Department of Biology, College of Education, University of Garmian, Kalar, KRG/Iraq; 2 School of Agriculture, Policy and Development, University of Reading, Reading, Berkshire, United Kingdom; United Arab Emirates University, UNITED ARAB EMIRATES

## Abstract

L-Ascorbic acid (ascorbate, Vitamin C) is an essential human micronutrient that is predominantly obtained from plants. It is known to work as the major antioxidant in plants, and it underpins several environmentally induced stresses due to its use as a co-factor by certain 2-oxoglutarate-dependent (2-OG) dioxygenases [2(OG)-dioxygenases]. It is important to understand the role of 2(OG)-dioxygenases in the biosynthesis of ascorbate. The present study examined contents of ascorbate and protein-protein interaction in nine T-DNA mutants of *Arabidopsis* containing an insert in their respective (2-OG) dioxygenase genes (*At1g20270*, *At1g68080*, *At2g17720*, *At3g06290*, *At3g28490*, *At4g35810*, *At4g35820*, *At5g18900*, *At5g66060*). In this study, the amount of ascorbate in five of the mutants was shown to be almost two-fold or more than two-fold higher than in the wild type. This result may be a consequence of the insertion of the T-DNA. The prediction of possible protein interactions between 2(OG)-dioxygenases and relevant ascorbate-function players may indicate the oxidative effects of certain dioxygenase proteins in plants. It is expected that certain dioxygenases are actively involved in the metabolic and biosynthetic pathways of ascorbate. This involvement may be of importance to increase ascorbate amounts in plants for human nutrition, and to protect plant species against stress conditions.

## Introduction

Ascorbate is an essential human micronutrient that is predominantly obtained from plants. Early use of large dose of ascorbate is currently proven to provide a useful adjuvant therapy in moderate-severe patients with coronavirus (SARS-CoV-2) infection [1]. The potential benefit of increasing the level of L-ascorbic acid (ascorbate) in plants may enhance human nutrition, allow new metabolic engineering and improve the tolerance of crops under stress conditions [2,3]. Numerous pathways of ascorbate synthesis have been elucidated, and the details of such pathways are becoming more complete [4]. However, regulation of such pathways to control levels of ascorbate is still under investigation. Recent studies have discovered that a translational feedback mechanism can control ascorbate levels in plants; this discovery demonstrates a new insight into regulation, and represents an additional method to increase ascorbate levels [5]. It is known that ascorbate-related genes (*vtc1-5)* encode pivotal enzymes, which play important roles in the biosynthesis pathways of ascorbate in plants [6]. For example, a mutation in *vtc1-1*, results in defective hormonal homeostasis (auxin/ethylene homeostasis) and nitric oxide signalling. Such mutants are expected to be more tolerant to abiotic stresses as a consequence of containing higher amount of ascorbate [2,7].

Due to the protective role of ascorbate as an antioxidant [8,9], and the use of this antioxidant as a co-factor by 2(OG)-dioxygenases [10] these enzymes might contribute in several physiological responses to unfavourable conditions in plants [11,12]. It is known that ascorbate serves as a cofactor for 2(OG)-dioxygenases, enzymes that are involved in several physiological activities [3,13]. For example, they play an important role in the synthesis of hormones including, gibberellins, abscisic acid, ethylene and the degradation of hormones such as indole acetic acid (IAA). These enzymes are also involved in the synthesis of a wide range of secondary compounds, including glucosinolates and anthocyanins [14]. In light of this evidence, mutants containing a T-DNA insert in their respective 2(OG) dioxygenase genes might demonstrate inactivation of these genes. Such inactivation is expected to lead to the subsequent inhibition/activation of other genes that encode enzymes that are actively involved in the biosynthesis of ascorbate. This involvement might increase ascorbate levels and also improve the tolerance of plants against stress conditions. To examine this prediction, the present study was designed to determine the content of ascorbate in WT and nine homozygous *Arabidopsis* mutants containing a T-DNA insert in their respective 2(OG) dioxygenase genes.

## Material and methods

### Plant materials

Seeds of WT and nine T-DNA insert mutant lines [N671573 (insert in *At1g20270*), N668172 (*At1g68080*), N652869 (*At2g17720*), N679576 (*At3g06290*), N678627 (*At3g28490*), N338446 (*At4g35810*), N683883 (*At4g35820*), N666896 (*At5g18900*), N598611 (*At5g66060*)] of *A*. *thaliana* Columbia ecotype were obtained from the European *Arabidopsis* Stock Centre (http://arabidopsis.info/BasicForm). Prior to germination seeds were soaked in water and kept in the fridge at 4°C for 4 d (stratified) in order to break seed dormancy.

### Seed germination

Stratified seeds were grown and germinated in 6.5 x 6 cm mini-flower pots (2 seeds in each pot) containing potting growing medium (supplied from http://www.sinclairpro.com/) and topped with vermiculite, pots were maintained under controlled environmental conditions at 22°C and 60% Relative Humidity (RH) with a day/night cycle of 16/8 h (at 100 μmol m^-2^ s^-1^ light intensity) in the growth chambers (Fitotron plant growth chambers, Weiss Gallenkamp, UK).

This study was conducted using a complete randomised design (CRD) with three replicates (used as biological replicates) and plants from each replicate (used as technical replicates).

### Confirmation of genetic status of mutant lines

To confirm the genetic status (homozygous, heterozygous or wild type) of the underlying mutant lines, their genomic DNA (5 plants/mutant) was extracted using a DNeasy Plant Mini kit (http://www.qiagen.com). The PCR mixture (25 μl) contained 2 μl of DNA, 0.75 μl (0.3 μM) of each forward primer (or left border primer LBb1.3, 5'-ATTTTGCCGATTTCGGAAC-3' and NR80 5'-GCTGATACAAAAACAAAACAACGA-3' were used with SALK and GK insertions respectively) and reverse primer (S1 Table), 12.5 μl 2X BioMix PCR master mix (Bioline, UK,) and 9 μl TE water. The reaction mixtures were amplified in a GeneAmp PCR system 9700 (Applied Biosystems). The PCR products were separated using electrophoresis in 1% agarose gels supplemented with ethidium bromide 3 μl/70 ml. PCR products were visualized on a GelDoc-ItTS2 Imager (UVP) followed by capturing a clear image through GeneSnap (version 6.00.19) system (SynGene, UK). Finally, each homozygous plant/line was determined, labelled and seeds was collected from those plants for further investigations [15].

### Sample preparation

Seeds from each homozygous plant/line were grown and germinated according the protocol stated above. Then, samples of fresh leaves were taken from 3-week old plants (mutants and wild type) of similar morphology. 1 ml of a solution containing 0.1 M HCl and 1 mM EDTA was added to the fine-ground powder of each sample. This mixture was incubated for 5 minutes, after which the mixture was thawed and then centrifuged at 13 000 rpm for 10 min. The supernatant was filtered and transferred to a new 1.5 ml Eppendorf tube. All steps were conducted at 4°C in a dark room.

### Determination of ascorbate levels

**Fig 1:**
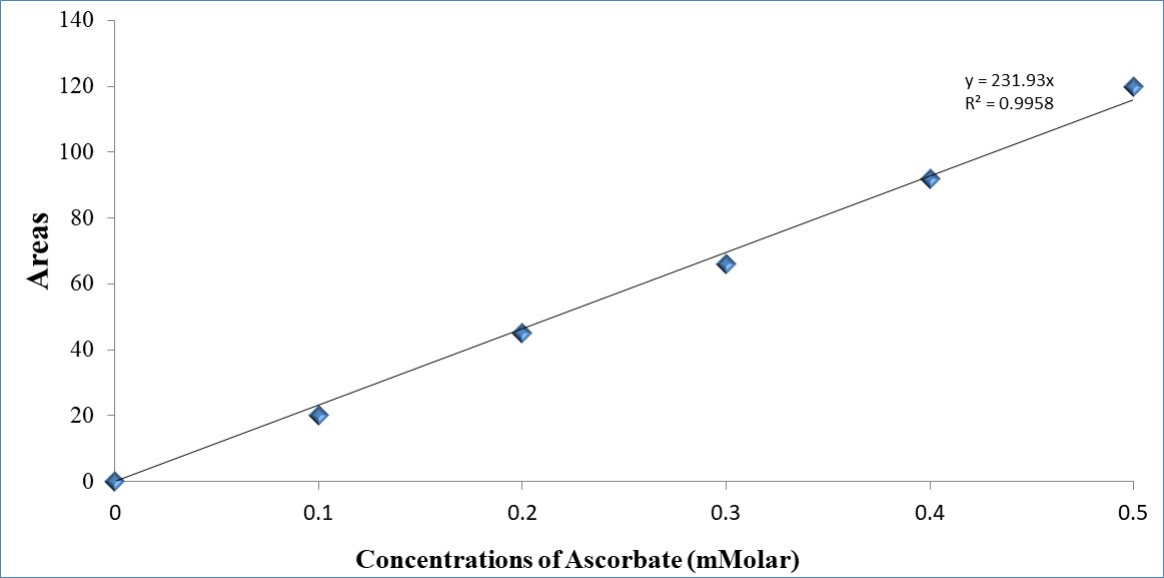
Standard curve for ascorbate dissolved in 1 ml of 0.1 M HCl and 1 mM EDTA.

Prepared samples were immediately used to measure total content of ascorbate using Capillary Electrophoresis (EC). Linear standards (0.1, 0.2, 0.3, 0.4, and 0.5) mM of L- ascorbate were dissolved in the same solvent and run in parallel with three technical replicates of each sample. All reads (Peak areas) were measured with the absorbance at 254 nm (S1 Fig). The measured area of each sample was converted to concentration using a linear equation generated from the intercept of standard values. The standard curve for ascorbate was generated with a linear intercept starting from zero over a broad range of concentrations up to 0.5 μMol. The value of coefficient of determination (R^2^ = 0.9958) was highly significant and the generated linear equation was used to calculate and normalise the amount of total ascorbate in WT and the nine homozygous mutant lines (Fig 1).

Furthermore, to quantify the amount of ascorbate, the measured peak area of each replicate/mutant and WT was converted to concentration using the linear equation (Y = 231.93X) in which Y refers to the concentration of ascorbate and X indicates area measurement of each experimental unit (replicate). The amount of ascorbate was adjusted to be expressed as μMolar/g fresh weight. Results were analysed using a CRD with one-way ANOVA and means compared using the Duncan’s multiple range test.

### Protein-protein interactions

Protein–protein interactions in association with physical and biological activities were investigated between underlying dioxygenases and enzymes involved in ascorbate biosynthesis of ascorbate. We used the latest version of String (11.0) protein–protein interaction networks (http://string-db.org/). This software is regarded as a valuable means to study comprehensive functional associations and integrations between proteins of 5090 organisms including *A*. *thaliana* [16].

## Results

### Ascorbate levels in the different mutant lines

**Fig 2:**
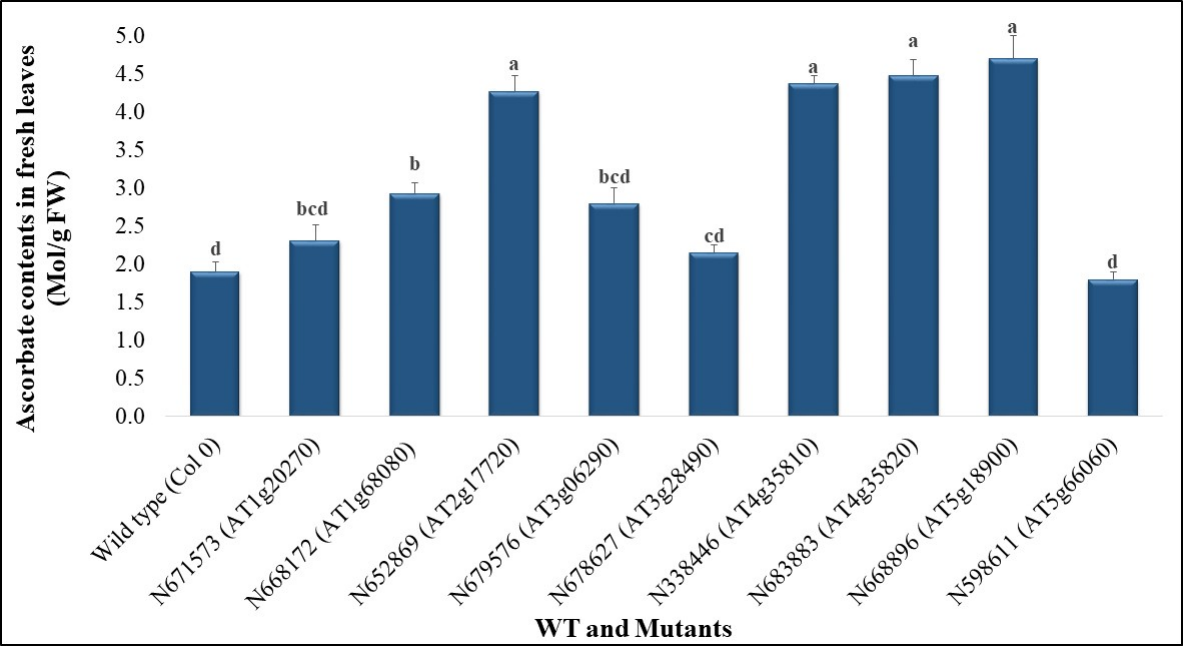
The content of ascorbate in WT and nine mutant lines. The values are expressed as means ± SE (n = 9), (P < 0.05). Means followed by the same letters within a column do not differ significantly from each other according to Duncan’s multiple range tests. FW denotes fresh weight.

The results indicate that five mutant lines (Fig 2) showed significantly higher levels of ascorbate compared to the wild type control (WT). The maximal ascorbate content (4.70± 0.3 Mol/g FW) was detected in the N666896 mutant. This amount was significantly higher by more than two fold compared with the WT (1.9± 0.13 Mol/g FW). Moreover, the ascorbate levels in the other three mutants (N652869, N338446 and N683883) was also significantly different compared to the WT. The amount of ascorbate in the latter mutants was significantly higher by more than two fold (4.27±0.2, 4.37± 0.1 and 4.48± 0.2 Mol/g FW respectively) compared with the WT. Despite giving a slightly higher level of ascorbate, the N668172 mutant showed a significantly higher content of ascorbate (2.93± 0.14 Mol/g FW) than the WT.

However, the results indicate that in four mutant lines, N598611, N678627, N671573, and N679576, no significant differences in ascorbate contents were detected between them (1.8± 0.1, 2.15± 0.1, 2.31± 0.2, and 2.80± 0.2 Mol/g FW respectively) and the WT control (1.9± 0.13 Mol/g FW). Although the mutant N668172 showed a significant difference compared to the WT, no significant differences were observed between the latter mutant and both N671573 and N679576 mutants.

### Protein-protein interactions with relevant ascorbate-function proteins

**Fig 3:**
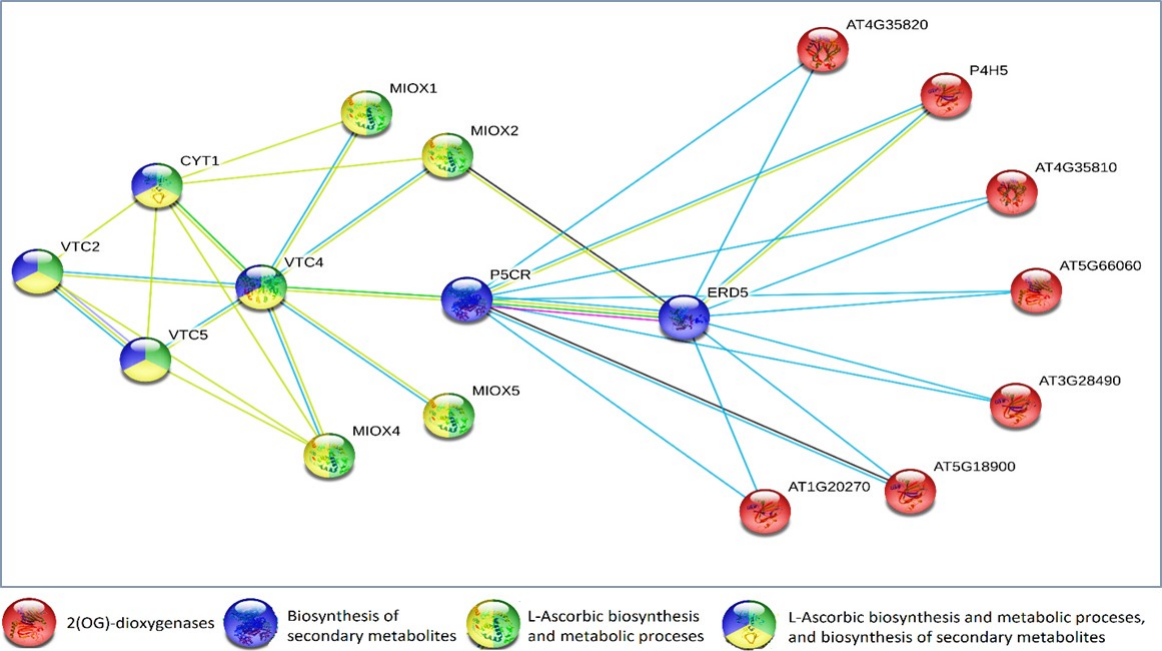
A prediction of protein-protein links between respective dioxygenases and other ascorbate-related proteins in *A*. *thaliana* (http://string-db.org/).

An analysis was conducted to determine whether the activities of respective dioxygenase proteins directly affect enzymes involved in ascorbate biosynthesis. These enzymes included:- CYT1 or VTC1, a GDP-mannose pyrophosphorylase/mannose-1-pyrophosphatase [17]; VTC2, a GDP-L-galactose phosphorylase 1 [18]; VTC3, a protein kinase and PP2C-like domain-containing protein [19]; VTC4, a L-galactose-1-phosphate phosphatase [20], and VTC5, a GDP-L-galactose phosphorylase [21]. The results of protein-protein interactions presented in Fig 3 show that indirect protein links are predicted between respective proteins and VTC1-5 in *A*. *thaliana*.

In this figure several interactions were identified; they included:- (I) Proline dehydrogenase 1 (EARLY RESPONSIVE TO DEHYDRATION 5: ERD5) that helps the recovery of plants from drought stress and is involved in the biosynthesis of proline [22,23], (II) PYRROLINE-5- CARBOXYLATE REDUCTASE (P5CR), an enzyme linked to the stress-induced accumulation of proline [24], (III) MYO-INOSITOL OXYGENASE 2 (MIOX2), an enzyme involved in ascorbate biosynthesis in plants. VTC1 (CYT1) was shown to interact with dioxygenases by only one textmining line (‘automated text-mining is applied to uncover statistical and/or semantic links between proteins, based on Medline abstracts and a large collection of full-text articles’) [25]. However, neither VTC2 nor VTC5 had any direct links with dioxygenases. On the other hand, VTC4 was intermediate between both VTC2 and VTC5 with dioxygenase by two steps through both MIOX2 and ERD5. These combinations were based on the textmining and database lines. Moreover, in this protein–protein interaction study there was no obvious association between VTC3 and respective proteins.

**Fig 4:**
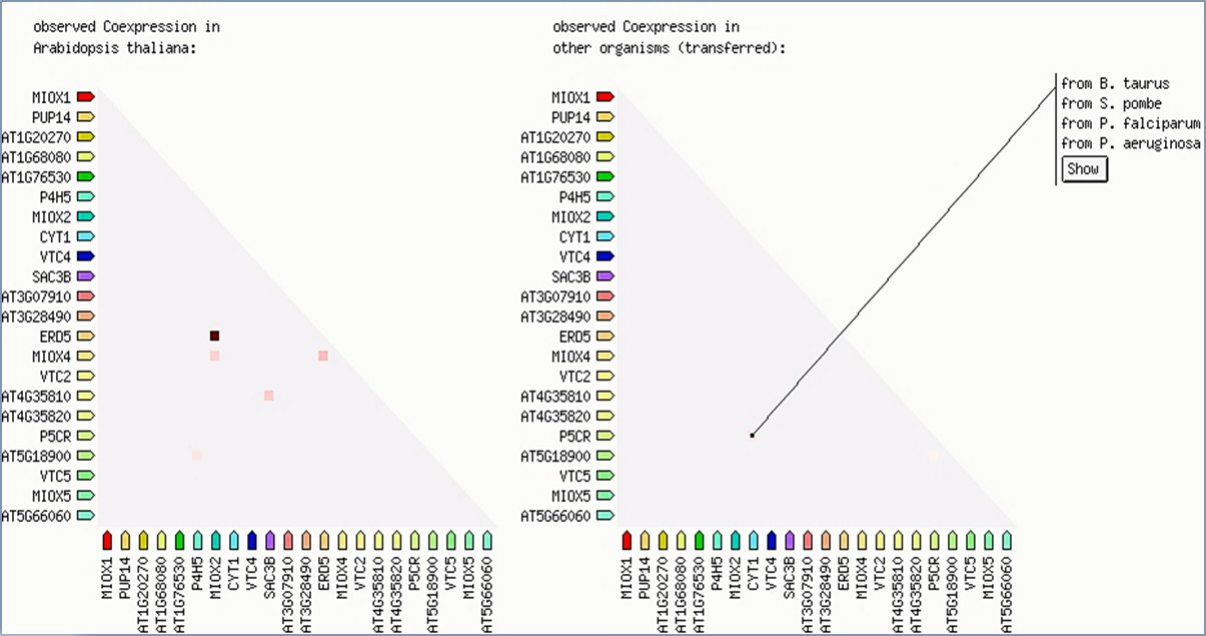
A prediction of co-expression links between respective dioxygenase genes and other ascorbate-related genes in *A*. *thaliana* and other organisms (http://string-db.org/).

While various protein-protein interactions were predicted between underlying proteins and VTC, MIOX2, ERD5 and P5CR, no gene co-expression was predicted between latter proteins and selected dioxygenases (Fig 4).

## Discussion

### Ascorbate levels

Based on their ascorbate levels, mutants could be divided into two groups. The first group comprise five mutant lines that had significant increases in the level of ascorbate in comparison with WT. This increase may be linked to the down-regulation of respective genes in mutants leading to the activation of other genes that encode enzymes involved in the biosynthetic pathways of ascorbate [11,26,27].

The second group comprise four mutants that all showed an increased amount of ascorbate compared to the WT, although these increases were not significant. In summary, these results may be linked to the suppression of respective genes in these mutants leading to the activation of other genes that encode enzymes involved in the biosynthetic pathways of ascorbate [26,27].

The ascorbate synthesis route in plants is principally involves the L-galactose pathway, which is also known as the Wheeler–Smirnoff pathway [28]. The enzyme GDP-L galactose phosphorylase (GGP, also known as VTC4) has an important role and is responsible for the sixth enzymatic step of this pathway. Numerous studies have documented that the overexpression of the GGP gene consistently leads to a two–six fold increases in ascorbate levels in a wide range of species including *A*. *thaliana* and [3,29]. Given the above premises, a link among 2(OG)-dioxygenase genes and the GGP gene expression is possible, whereby metabolic reprogramming occurs in the mutants studied. The present results indicate that suppression of certain 2(OG)-dioxygenase genes may possibly up-regulate expression of the GGP gene and subsequently increase the ascorbate level in plants.

Although ascorbate has an active role as a co-factor of 2(OG)-dioxygenase family enzymes, it is also a natural substrate of ascorbate peroxidases in plants [30]. These latter enzymes are important players in regulating levels of cellular reactive oxygen species (ROS) under stress conditions [31] and in addition, 2(OG)-dioxygenase are thought to act as direct sensors of oxygen and regulators of hypoxia in both plants, and animals. Specifically, ROS metabolism consists of a complex network, which interacts closely with hormonal signalling systems, and allows plants a subtle developmental regulation against biotic and abiotic stress responses [32,33]. In light of the results from the present study, it is expected that selected mutants found to contain a high amount of ascorbate may be more tolerant to abiotic stress including ozone (O_3_), high salinity and drought stress [2,6,11]. Despite the expectation of that selected dioxygenases are involved in the metabolic and biosynthetic pathways of ascorbate, to date there is no experimental evidence to confirm this idea yet. However, to further investigate this concept and to inform future studies, we studied protein-protein interactions between specific dioxygenase enzymes and ascorbate-function proteins.

### Protein-protein interactions with relevant ascorbate-function proteins

Analysis of Protein-protein interactions predicted several interactions with gene products known to be involved in biosynthetic and metabolic processes of ascorbate. The prediction of protein interactions between VTC, MIOX and the selected 2(OG)-dioxygenases may indicate the latter enzymes are involved in regulatory mechanisms during embryonic development, germination, fruit ripening and in the regulation of auxins, which comprise an important class of plant hormone. These regulatory mechanisms are known to be involved in ascorbate biosynthesis [34–37]. Possible protein interaction between P5CR, VTC and underlying 2(OG)-dioxygenases, may be a signal of the active role of such dioxygenases in protecting plants against drought and salinity. These expectations derive from previous studies demonstrating the role of P5CR in the biosynthetic pathways of proline, a widely distributed osmolyte that protect plants from stress conditions [38,39]. ERD5 is another protein predicted to be intermediated between VTC and underlying proteins. These interactions indicate the oxidative effect of dioxygenase proteins in the epigenetic changes of plants; these changes may serve as adaptive mechanisms to environmentally induced abiotic stress. This expectation derives from findings that reveal the involvement of ERD5 in the adaptation of *A*. *thaliana* accessions to cold, drought and salt tolerance [40]. It has also been shown that one of the mechanisms underlying the early adaptation of plants to salt stress is associated with an epigenetic mark [23].

In conclusion, evidence presented here strongly suggest that products of certain dioxygenase genes are expected to be actively involved in the metabolic and biosynthetic pathways of ascorbate [41]. This involvement may be of importance in future efforts to increase ascorbate amounts in plants for human nutrition [1–3,6,11,42], and to protect crop species against unfavourable conditions including drought, cold, light, O_3_ and salinity [7,43].

## Supporting information

S1 Fig Showing area measurements of ascorbate level (at approximately 6 min.) in WT and nine mutants. (ZIP)

S1 Table List of primer sequences for detecting DNA insertion in mutant lines of *Arabidopsis*. (DOCX)
