# Supplementary figures and images for: 2-oxoglutarate-dependent dioxygenases: A renaissance in attention for ascorbic acid in plants
Source: PLoS One. 2020 Dec 8;15(12):e0242833. doi: 10.1371/journal.pone.0242833 (PMC7723244; doi:10.1371/journal.pone.0242833)

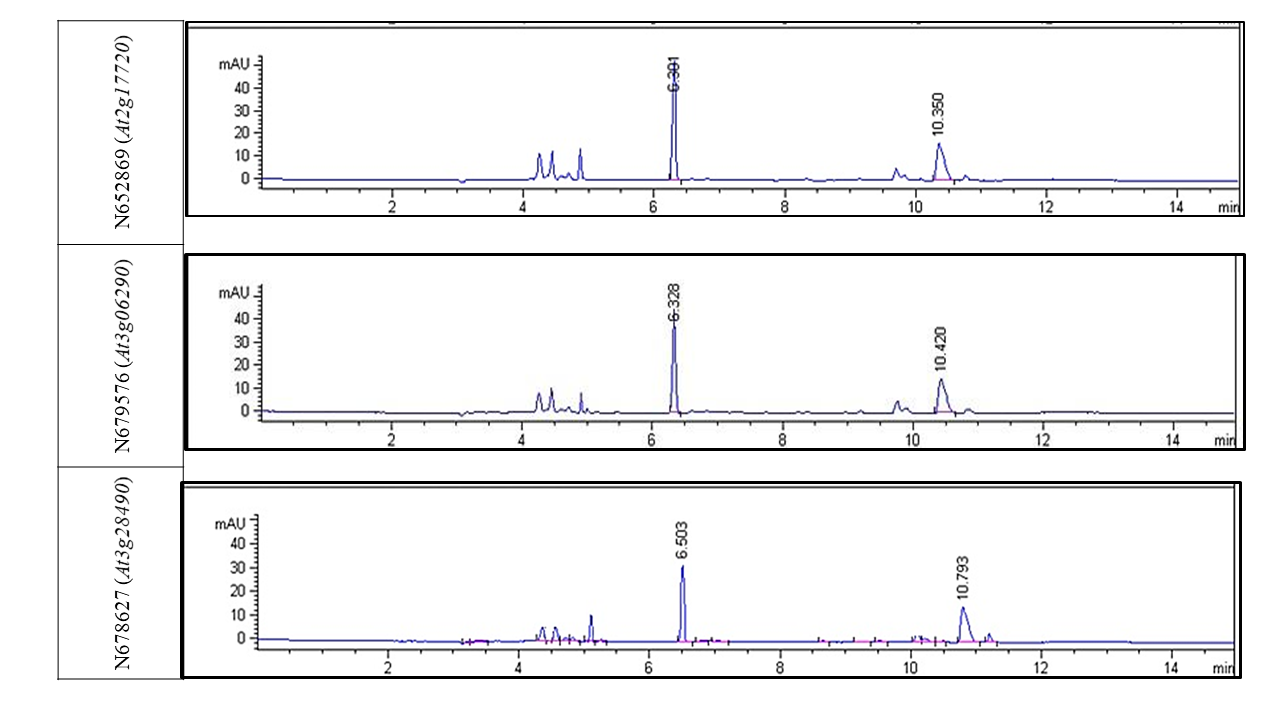

Supplement: S1 Fig — (ZIP) [file pone.0242833.s001.zip › PACE Corrected/2.tif]

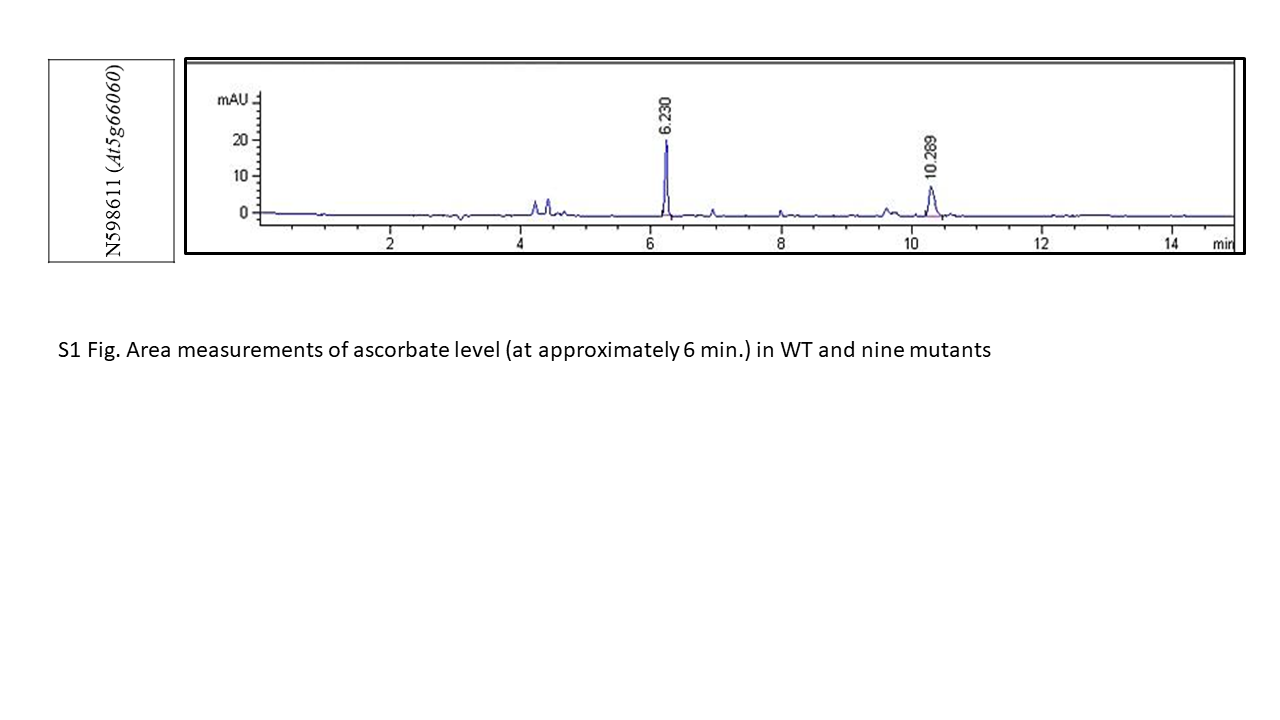

Supplement: S1 Fig — (ZIP) [file pone.0242833.s001.zip › PACE Corrected/4.tif]

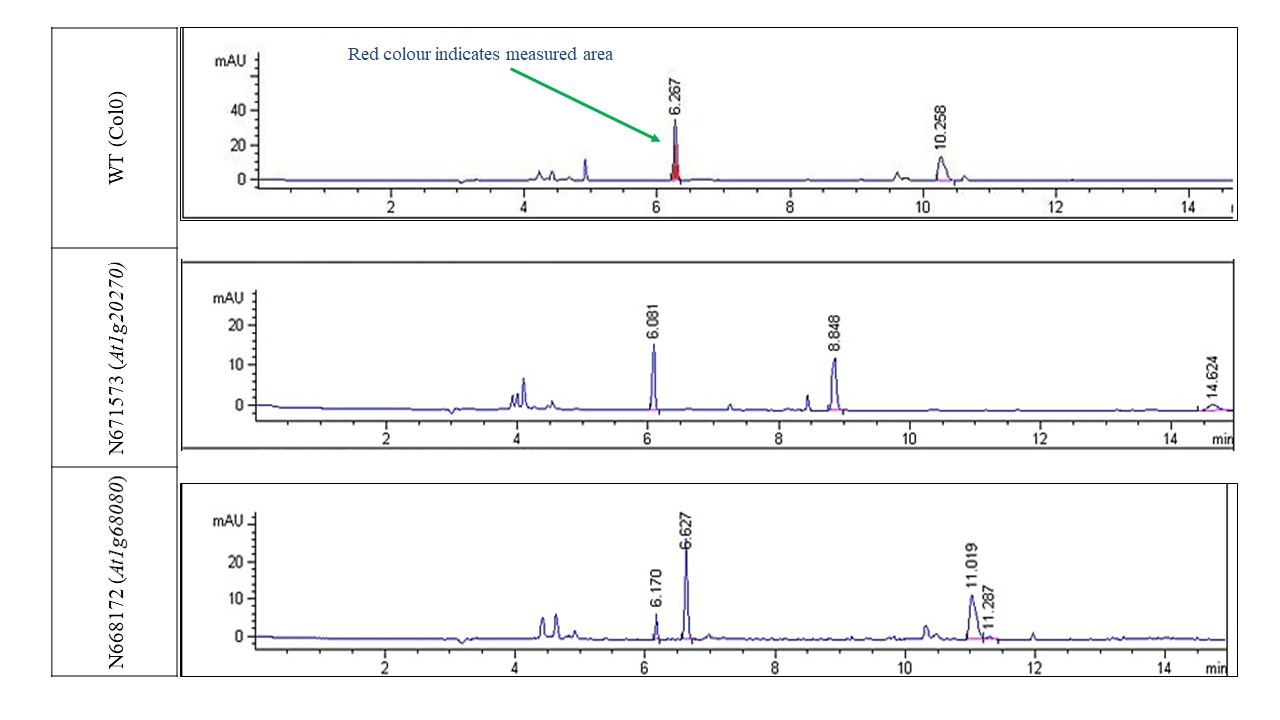

Supplement: S1 Fig — (ZIP) [file pone.0242833.s001.zip › PACE Corrected/1.tif]

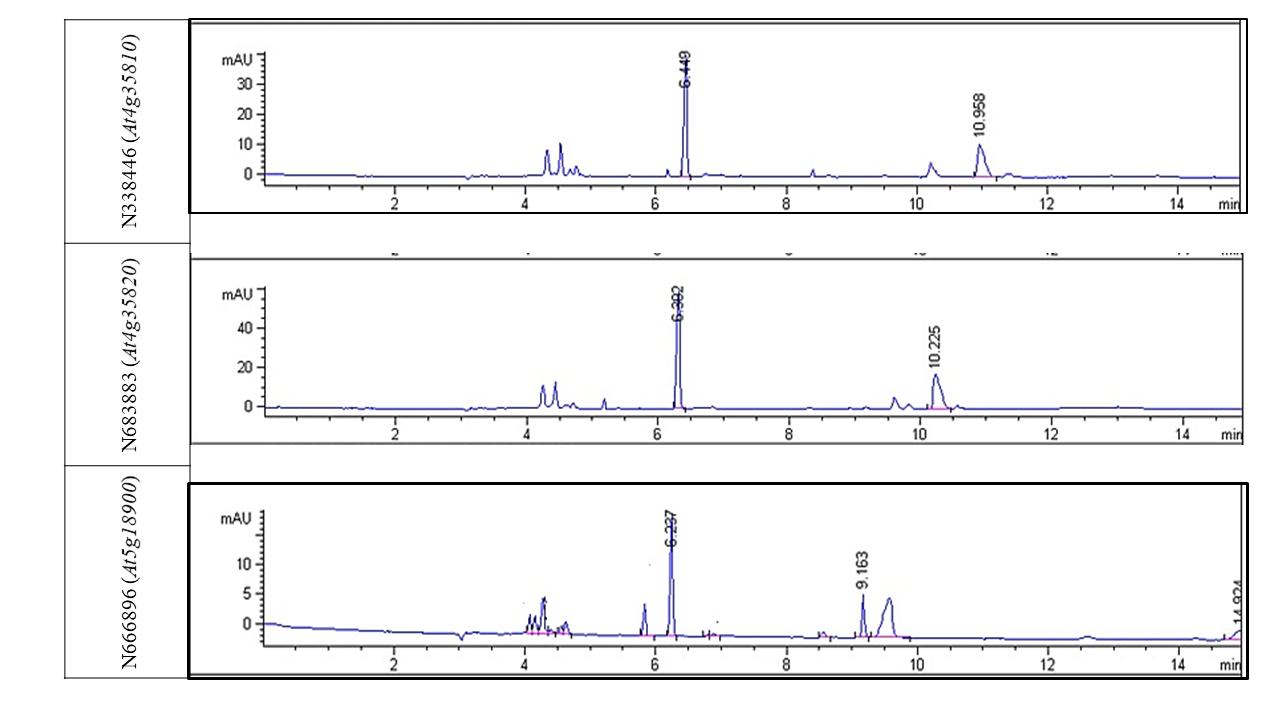

Supplement: S1 Fig — (ZIP) [file pone.0242833.s001.zip › PACE Corrected/3.tif]
