# Supplementary material for: 2-oxoglutarate-dependent dioxygenases: A renaissance in attention for ascorbic acid in plants
Source: PLoS One. 2020 Dec 8;15(12):e0242833. doi: 10.1371/journal.pone.0242833 (PMC7723244; doi:10.1371/journal.pone.0242833)
Supplement: S1 Table — (DOCX) [file pone.0242833.s002.docx]

S1 Table. List of primer sequences for detecting DNA insertion in mutant lines of *Arabidopsis*

| **Ensemble Genes ID** | **NASC Codes** | **Gene clones** | **5’🡪 3’ primer sequence (LP)** | **TM (C°)** | **5’🡪 3’ primer sequence (RP)** | **TM (C°)** |
| --- | --- | --- | --- | --- | --- | --- |
| ***At1g20270*** | N671573 | SALK_076446.55.75.x* | CCACAAACTCAATGGTATCGG | 60.23 | GAGACCAGTGGACTGAAGTGC | 59.90 |
| ***At1g68080*** | N668172 | SALK_044417.41.80.x | AAAGGAAGCCCGTTTAACAAG | 59.65 | TCTCTCTCACCTCATTGCCAC | 60.40 |
| ***At2g17720*** | N652869 | SALK_152869.16.30.x | CATTTTGAGAGCTCGTTCCAC | 59.87 | AGTTATTTCTTGGGAGCCTCG | 59.73 |
| ***At3g06290*** | N679576 | SALK_042128.56.00.x | CAAAGAGCAAAATTCGCACTC | 60.01 | TAAAGCAACTCGCAGCTTCTC | 59.92 |
| ***At3g28490*** | N678627 | SALK_067682.42.20.x | TGCCACACAATATTTTTCTGAAG | 59.18 | TACCCTCAGGAAGAAAGGTCC | 59.56 |
| ***At4g35810*** | N338446 | GK-859B10.01** | TTCGACGCA AACTTTCACAG | 59.00 | ATATTGACCATCATACTCATTGC | 59.00 |
| ***At4g35820*** | N683883 | SALK_023179.31.3 5.x | ATAAAAGGAGGATCAGACGGC | 59.57 | TTGAAATCCGTCAAAGTGAGG | 60.10 |
| ***At5g18900*** | N666896 | SALK_102582.48.00.x | ATACACAAACGCCCTGAACAC | 59.91 | AGCGAGAAAACTCCAAACTCC | 59.88 |
| ***At5g66060*** | N598611 | SALK_098611.42.75.x | TTGGTTTCACAGACAACCCTC | 60.00 | GCGAAGATGATTCAAAGAACG | 59.84 |

[*http://signal.salk.edu/cgi-bin/atta?JOB=APPENDIX&QUERY=PrimerL](http://signal.salk.edu/cgi-bin/atta?JOB=APPENDIX&QUERY=PrimerL)

[**https://www.gabi-kat.de/db/genotyping_details.php?lineid=859B10&genecode=At4g35810](https://www.gabi-kat.de/db/genotyping_details.php?lineid=859B10&genecode=At4g35810)

LP= left or forward primer, RP= Reverse primer
